# Leukotrienes Are Dispensable for Vaginal Neutrophil Recruitment as Part of the Immunopathological Response During Experimental Vulvovaginal Candidiasis

**DOI:** 10.3389/fmicb.2021.739385

**Published:** 2021-11-17

**Authors:** Junko Yano, David J. White, Anthony P. Sampson, Floyd L. Wormley, Paul L. Fidel

**Affiliations:** ^1^Center of Excellence in Oral and Craniofacial Biology, School of Dentistry, Louisiana State University Health Sciences Center, New Orleans, LA, United States; ^2^Department of Genitourinary Medicine, University Hospitals Birmingham NHS Foundation Trust, Birmingham, United Kingdom; ^3^Clinical and Experimental Sciences, Faculty of Medicine, University of Southampton, Southampton, United Kingdom; ^4^Department of Biology, Texas Christian University, Fort Worth, TX, United States

**Keywords:** vulvovaginal candidiasis, *Candida albicans*, immunopathology, leukotriene receptor antagonists, inflammatory responses

## Abstract

Recruitment of polymorphonuclear neutrophils (PMNs) into the vaginal lumen is the hallmark of an acute immunopathologic inflammatory response during vulvovaginal candidiasis (VVC) caused by *Candida albicans.* Recurrent VVC (RVVC) remains a chronic health burden in affected women worldwide despite the use of antifungal therapy. Based on the role leukotrienes (LTs) play in promoting inflammation, leukotriene receptor antagonists (LTRAs) targeted for LTB_4_ (etalocib) or LTC_4_, LTD_4,_ and LTE_4_ (zafirlukast or montelukast) have been shown to reduce inflammation of epithelial tissues. An open-label pilot study using long-term regimens of zafirlukast in women with RVVC indicated the potential for some relief from recurrent episodes. To investigate this clinical observation further, we evaluated the effects of LT antagonistic agents and LT deficiency on the immunopathogenic response in a mouse model of VVC. Results showed that mice given daily intraperitoneal injections of individual LTRAs, starting 2days prior to vaginal inoculation with *C. albicans* and continuing through 14days post-inoculation, had no measurable reduction in PMN migration. The LTRAs were also ineffective in reducing levels of the hallmark vaginal inflammatory markers (S100A8, IL-1β) and tissue damage (LDH) associated with the immunopathogenic response. Finally, LT-deficient 5-lipoxygenase knockout mice showed comparable levels of vaginal fungal burden and PMN infiltration to wild-type mice following inoculation with a vaginal (ATCC 96113) or laboratory (SC5314) *C. albicans* isolate. These results indicate that despite some clinical evidence suggestive of off-target efficacy of LTRAs in RVVC, LTs and associated signaling pathways appear to be dispensable in the immunopathogenesis of VVC.

## Introduction

Vulvovaginal candidiasis (VVC), predominantly caused by *Candida albicans*, is a common fungal infection in women of reproductive age, affecting approximately 75% of the otherwise healthy female population at least once during their lifetime (Sobel, 2007; Yano et al., 2019). Fulminant VVC is typically described as itching, burning, redness of the vulva, and vaginal mucosa accompanied by cottage cheese-like vaginal discharge (Sobel, 1992). Despite the high incidence worldwide, current therapies for VVC are largely limited to topical or oral antifungal agents, primarily azoles, most of which are effective for treatment of acute episodes *via* fungistatic, not fungicidal, mode of action (Sobel, 1992). Incomplete clearance of infection can result in a rapid relapse, causing prolongation of debilitating symptoms and healthcare burden especially in those susceptible to recurrent VVC (RVVC), defined as ≥4 episodes of VVC per year (Sobel, 1997).

Historically, susceptibility to VVC/RVVC was believed to be attributed to systemic and local immunodeficiencies similar to other forms of candidiasis (Fidel, 2002; Yano et al., 2012), in which infections occur due to compromised T-cell immunity (oropharyngeal candidiasis; Pirofski and Casadevall, 2009; Fidel, 2011; Gaffen et al., 2011), immunosuppression from neutropenia (candidemia; Walsh and Gamaletsou, 2013; Mohammadi and Foroughifar, 2016), or direct invasion of the organisms into the bloodstream through abdominal trauma or indwelling catheters (disseminated candidiasis; Vonk et al., 2006; Koh et al., 2008). However, more recent findings from a live challenge study and a series of work using a mouse model revealed that an acute inflammatory response by polymorphonuclear neutrophils (PMNs) occurs during vaginal infection with strong correlation with severity of VVC symptomatology (Fidel et al., 2004; Yano et al., 2010; Peters et al., 2014). Subsequent studies in mice identified paramount mediators of PMN migration and its positive feedback response, namely, IL-1β and S100A8, as hallmarks of symptomatic infection (Yano et al., 2014; Willems et al., 2018). In addition, the inflammatory response is also associated with tissue damage as measured by lactate dehydrogenase (LDH; Yano et al., 2014; Willems et al., 2018). Hence, VVC/RVVC is now considered an immunopathology driven by a strong PMN response to *C. albicans* (Yano et al., 2018). After nearly three decades of extensive research, however, mechanisms of protection against *C. albicans* vaginal infection are not well understood.

Despite the robust recruitment and abundance, PMNs have no apparent contribution to fungal clearance in the vaginal environment while fully retaining pro-inflammatory properties (Yano et al., 2010). A recent study using mouse strains exhibiting susceptible vs. resistant conditions of experimental VVC indicated that vaginal heparan sulfate (HS), which is present at high levels under the estrogen-responsive state, serves as a competitive ligand for a PMN cell surface receptor Mac-1, thereby interfering with the binding to *C. albicans* Pra-1 (Yano et al., 2017). Hence, the PMNs fail to kill *C. albicans* but retain the activated inflammatory state in the vaginal mucosa. Given the novel mechanism of vaginal PMN dysfunction, termed “Neutrophil Anergy” (Yano et al., 2018), therapeutic approaches should focus on preventing or reducing VVC immunopathology so as to eliminate the symptomatic condition and reduce recurrent episodes.

Leukotrienes (LTs) are a group of eicosanoid inflammatory mediators derived from arachidonic acid *via* the 5-lipoxygenase (5-LO) pathway (Dixon et al., 1990). The enzyme 5-LO is an essential component of the biosynthesis of all LT products in various leukocytes, including neutrophils, and deletion of *5-LOX* results in complete LT deficiency in mice (Reid et al., 1990). In response to inflammatory stimuli, such as infections, arachidonic acid is liberated from the cell membrane and converted *via* the sequential actions of 5-LO and downstream enzymes into LTB_4_ or cysteinyl leukotrienes (cys-LTs) comprising of LTC_4_, LTD_4_, and LTE_4_ (Dixon et al., 1990). LTB_4_ is known to be a potent mediator of neutrophil chemotaxis into target tissues, and excess production can lead to neutrophil-mediated pathology as seen in type-2 diabetes and rheumatoid arthritis (Chen et al., 2006; Mathis et al., 2007; Spite et al., 2011). Similarly, cys-LTs are produced by a variety of activated leukocytes, including neutrophils, mast cells, and eosinophils, and are predominant effectors in pathogenesis of asthma (Niimi, 2013). Various LT receptor antagonists (LTRAs) have been developed, of which cys-LTRAs zafirlukast and montelukast are best characterized, currently approved drugs for the prevention and treatment of asthma (Suissa et al., 1997; Reiss et al., 1998). Other LTRA agents include etalocib, a selective LTB_4_ receptor antagonist, that has been under investigation (Bhatt et al., 2017).

Intriguingly, an open-label pilot study of zafirlukast indicated that regimens with 20mg twice daily for 24weeks provided symptom relief in women with RVVC (White et al., 2004). Considering the extensive efforts required for development of novel therapeutics, drug repurposing has tremendous advantages in comparison with traditional approaches to drug discovery. Therefore, the purpose of this study was to evaluate the effects of LTRA agents (etalocib, zafirlukast, and montelukast) and LT deficiency (*5-LOX* knockout mice) on the vaginal immunopathogenic response in a mouse model of VVC.

## Materials and Methods

### Mice

Female C3H/HeN mice at 6–8weeks old of age were purchased from Charles River Laboratories and used throughout the drug studies. Breeders deficient in 5-lipoxygenase (*5-LOX* ^−/−^) were provided by Dr. Floyd Wormley (Texas Christian University). Females at 5 to 8weeks of age from the breeding colony were used in parallel with age-matched C57BL/6 (wild-type). All animal protocols were reviewed and approved by the institutional animal care and use committee (IACUC) of the LSU Health-New Orleans.

### *Candida albicans* Strains

*Candida albicans* strains ATCC 96113 (a clinical vaginal isolate) or SC5314 (a standard laboratory strain) were used throughout the studies. Both strains were grown in yeast extract peptone-dextrose (YPD) broth for 18h at 30°C with shaking at 200rpm to reach a stationary-phase culture. Following incubation, the *C. albicans* yeast cells were washed three times in sterile phosphate-buffered saline (PBS) and enumerated on a hemocytometer.

### Vaginal *C. albicans* Inoculation

Intravaginal inoculation with *C. albicans* in mice was conducted as previously described (Yano and Fidel, 2011). Briefly, mice were administered 0.1mg β-estradiol 17-valerate (Sigma) dissolved in 100μl sesame oil (Sigma) by subcutaneous injection 72h prior to inoculation. Estrogen injections were repeated weekly until the end point to maintain the pseudo-estrous state in the mice. Estrogen-treated mice were intravaginally inoculated by introducing 20μl of PBS containing *C. albicans* 96,113 (5×10^4^) or SC5314 (5×10^6^) blastoconidia into the vaginal lumen. Groups of 4–10 mice (drug studies) or 5–15 mice (knockout studies) were evaluated longitudinally at designated time points post-inoculation.

### Leukotriene Receptor Antagonists

Groups of estrogen-treated, inoculated mice were administered leukotriene receptor antagonists (LTRAs) that target activity of respective leukotriene products of the 5-LO pathway (Supplementary Figure 1). Specifically, montelukast and zafirlukast were selected to examine the effect of cysteinyl leukotrienes LTC_4_, LTD_4_, and LTE_4_, and etalocib was used to target for LTB_4_ activity. All drugs were first dissolved in DMSO and further diluted in sterile PBS. Drugs were administered to the mice intraperitoneally using 100μl of each drug solution at the concentration of 20mg/kg (montelukast), 10mg/kg (zafirlukast), 1mg/kg (etalocib, also known as LY293111), or DMSO alone, once daily for 16days beginning 2days prior to vaginal inoculation. Initial dose responses were used in pilot studies based on experience of our co-authors and the literature (Oruc et al., 2004; Ghorbanzadeh et al., 2016; Li et al., 2018). Based on initial negative results, the highest concentrations possible for each drug were employed based on the limits of solubility in DMSO. LTRA drugs were purchased from Cayman Chemical (etalocib) or Sigma (zafirlukast/montelukast).

### Vaginal Lavage and Fungal Burden

Under anesthesia by isoflurane inhalation (longitudinal time points) or upon euthanasia (end point), vaginal lavage was performed using 100μl of sterile PBS with repetitive aspiration. Aliquots from recovered lavage fluids were removed to determine fungal burden and PMN quantification. The supernatants of the remaining fluids were stored at −80°C until use. To assess vaginal fungal burden, serial dilutions of the lavage fluid were cultured on Sabouraud-dextrose agar plates (BD diagnostics) supplemented with gentamycin (Invitrogen). After incubation for 24h at 37°C, colonies were enumerated, and results were expressed as CFU/100μl lavage fluid.

### PMN Quantification

Smear preparations of 10μl vaginal lavage fluid collected at each time point were stained using the Papanicolaou technique (Pap smear). PMNs, if present, were identified by their characteristic tri-lobed nuclear morphology and were the predominant infiltrating leukocytes during *C. albicans* vaginal infection as previously reported (Fidel et al., 2004; Yano et al., 2010). PMNs were enumerated in 5 nonadjacent fields per mouse by light microscopy using a 40× objective, and the mean values were used for data analyses.

### IL-1β and S100A8 ELISAs

Concentrations of IL-1β and S100A8 in vaginal lavage fluids were determined by a standard ELISA according to the instructions of the manufacturer (R&D Systems). Briefly, enzyme immunoassay (EIA) plates (Corning) were coated with monoclonal rat anti-mouse IL-1β or S100A8 antibodies overnight. After blocking of the nonspecific sites (1% bovine serum albumin) and washing with ELISA wash buffer (0.05% Tween 20-PBS), dilutions of lavage fluid supernatants (at 1:10 for IL-1β and 1:100~1,000 for S100A8) and serially diluted protein standards were added and incubated overnight at 4°C. Captured proteins were labeled with biotinylated polyclonal goat anti-mouse IL-1β or S100A8 antibodies followed by streptavidin-horseradish peroxidase (HRP). The antibody-HRP complexes were detected by a tetramethylbenzidine-H_2_O_2_ substrate solution, and the absorbance was measured at 450nm on a Multiskan Ascent microplate reader. All samples were measured in duplicate. Values are expressed as pg./ml±standard errors of the means (SEM).

### LTB_4_ ELISA

Concentrations of LTB_4_ in vaginal lavage fluids were determined by a standard ELISA according to the instructions of the manufacturer (Cayman Chemical). Briefly, a combination of LTB_4_-acetylcholinesterase conjugate (LTB_4_-AChE Tracer), specific antiserum to LTB_4_, and neat vaginal lavage fluid supernatants or serially diluted LTB_4_ standards were added to a 96-well plate precoated with mouse anti-rabbit IgG and incubated overnight at 4°C. After incubation and washing, the amount of LTB_4_-AChE Tracer bound to the wells, which was inversely proportional to the concentration of free LTB_4_ in the lavage fluid samples, was enzymatically detected by AChE substrate (Ellman’s reagent). The reaction was measured spectrophotometrically at 412nm on a BioTek Synergy microplate reader. All samples were measured in duplicate. Values are expressed as pg./ml±standard errors of the means (SEM).

### Lactate Dehydrogenase Assay

Levels of lactate dehydrogenase (LDH) release by the vaginal mucosa were determined by a standard LDH assay per the instructions of the manufacturer (Promega). The activity of LDH in the supernatants of lavage fluids was measured by recording the rate of change in NADH concentrations after interaction with a colorimetric probe. Absorbance was read at 490nm on a BioTek Synergy microplate reader. All samples were measured in duplicate. Values are expressed as optical density at 490nm (OD_490_).

### Statistics

Experiments were conducted using 4 to 15 mice per group. The unpaired Student’s *t* test was used for analyses with comparisons made between experimental and control groups at each time point. Significant differences were defined at a confidence level where the *p* value was <0.05. All statistical analyses were performed using Prism software (Graph Pad).

## Results

### Treatment With LTB_4_ Receptor Antagonist Do Not Reduce Vaginal PMN Migration During *C. albicans* Infection

**Figure 1:**
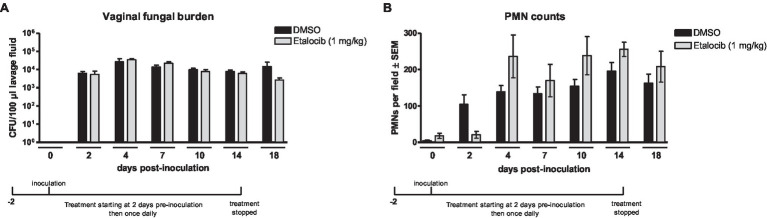
Treatment with a LTB_4_ receptor antagonist Etalocib does not reduce PMN migration to the vaginal cavity following inoculation with *C. albicans.* Estrogenized C3H mice were administered Etalocib (1mg/kg) or vehicle alone (10% DMSO) in a volume of 100μl by i.p. injection on 2days prior to inoculation then once daily for 16days. Mice were intravaginally inoculated with 5 × 10^4^ *C. albicans* 96,113. Vaginal lavage fluid was collected longitudinally over a period of 18days post-inoculation. **(A)** Vaginal fungal burden was assessed by quantitative plate counts at the indicated time points. **(B)** Vaginal cellular infiltrates were stained by the Pap smear technique and examined by light microscopy at x400 magnification. Number of PMNs were quantified in 5 nonadjacent fields per sample and averaged. Data represent cumulative results of 1 longitudinal experiment performed with 4–5 animals/group. Data were analyzed by using the unpaired Student’s t test comparing the drug-treated groups with vehicle control group at specific time points. CFU, colony-forming unit; PMN, polymorphonuclear leukocyte.

A strong inflammatory response by PMNs is a key event leading to VVC immunopathology both clinically and in mouse models (Fidel et al., 2004; Yano et al., 2010). Accordingly, we first sought to investigate whether LTB_4_, a potent neutrophil chemoattractant and activator of PMN-driven inflammation (Belanger et al., 2008; Lee et al., 2018), plays a role in vaginal immunopathology in response to *C. albicans.* To block LTB_4_ activity, mice inoculated with *C. albicans* (ATCC 96113, a vaginal isolate) were treated with etalocib, a selective LTB_4_ receptor antagonist, and longitudinally evaluated for fungal colonization and PMN migration during the course of infection. Results showed that mice receiving a daily dose of etalocib had similar high levels of vaginal fungal burden (Figure 1A) and PMN infiltrates (Figure 1B) throughout the observation period compared to the vehicle-alone control. Both drug-treated and control groups exhibited persistent fungal burden and PMN migration which remained unaffected following discontinuation of the treatments.

### Effects of cys-LT Receptor Antagonists on Vaginal PMN Migration During *C. albicans* Infection

**Figure 2:**
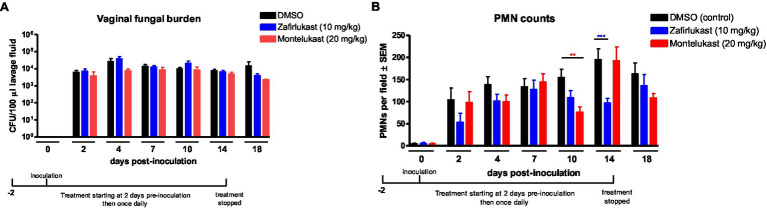
Treatment with cysteinyl leukotrienes receptor antagonists does not reduce PMN migration to the vaginal cavity following inoculation with *C. albicans.* Estrogenized C3H mice were administered Zafirlukast (10mg/kg), Montelukast (20mg/kg) or vehicle alone (10% DMSO) in a volume of 100μl by i.p. injection on 2days prior to inoculation then once daily for 16days. Mice were intravaginally inoculated with 5 × 10^4^ *C. albicans* 96,113. Vaginal lavage fluid was collected longitudinally over a period of 18days post-inoculation. **(A)** Vaginal fungal burden was assessed by quantitative plate counts at the indicated time points. **(B)** Vaginal cellular infiltrates were stained by the Pap smear technique and examined by light microscopy at x400 magnification. Number of PMNs were quantified in 5 nonadjacent fields per sample and averaged. Data represent cumulative results of 7 independent experiments performed with 5–10 animals/group. Data were analyzed by using the unpaired Student’s t test comparing the drug-treated groups with vehicle control group at specific time points. ^**^p<0.01; ^***^p<0.001. CFU, colony-forming unit; PMN, polymorphonuclear leukocyte.

Based on symptomatic relief observed in the previous open-pilot study employing a cys-LT-receptor antagonist (White et al., 2004), we tested whether antagonists to cys-LTs, zafirlukast and montelukast, could modulate the immunopathogenic response in inoculated mice. While vaginal fungal colonization was similar in all animals (Figure 2A), montelukast- and zafirlukast-treated mice had significantly reduced vaginal PMN migration 10 or 14 days post-inoculation, respectively (Figure 2B). In both cases, however, PMNs returned to control levels within 2days.

### Montelukast and Zafirlukast Do Not Alleviate Vaginal Inflammation During *C. albicans* Infection

**Figure 3:**
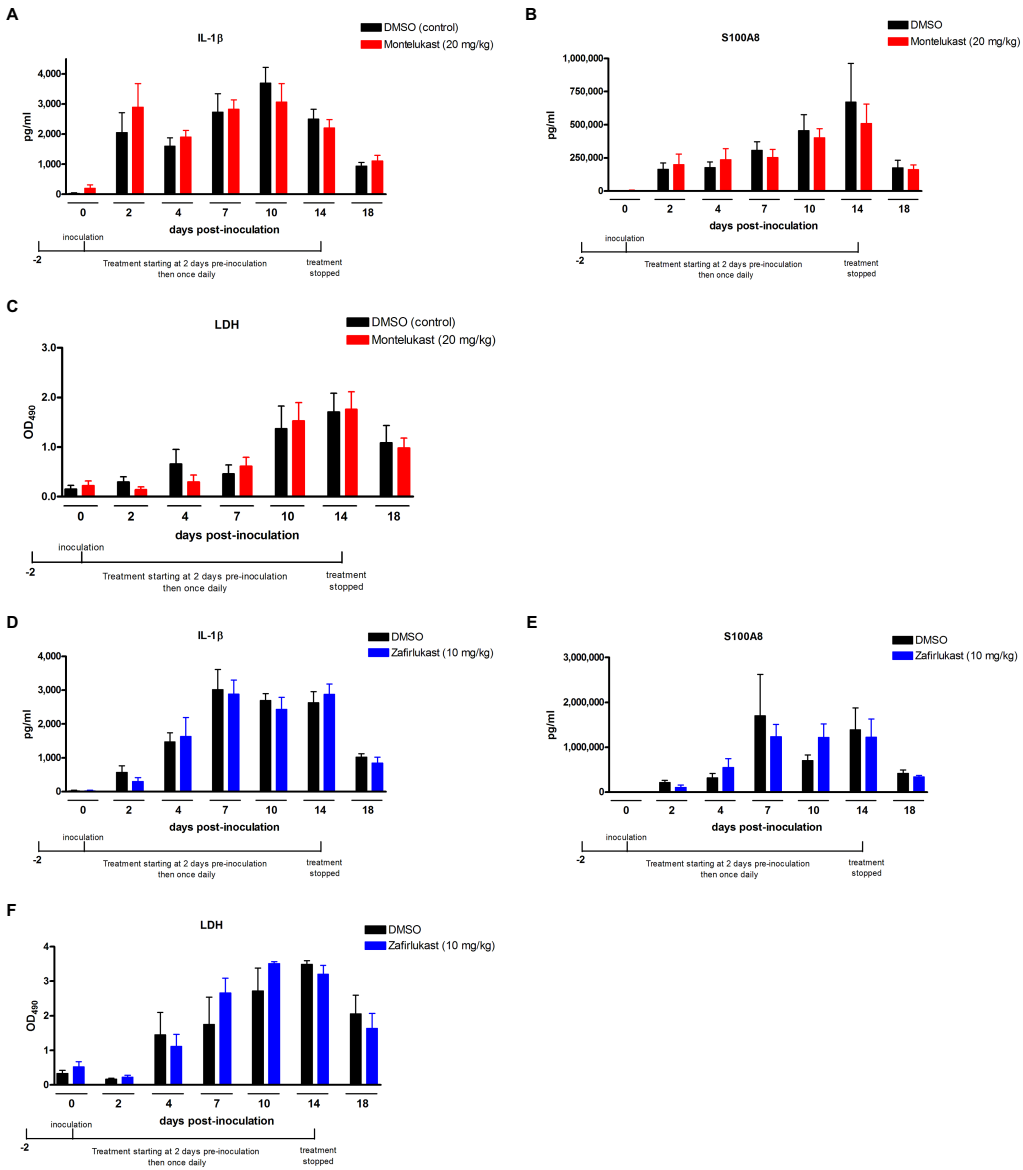
Treatment with leukotriene receptor antagonists does not alter the production of inflammatory mediators and vaginitis-associated tissue damage in the vaginal mucosa following inoculation with *C. albicans.* Estrogenized C3H mice were administered Zafirlukast (10mg/kg), Montelukast (20mg/kg) or vehicle alone (10% DMSO) in a volume of 100μl by i.p. injection on 2days prior to inoculation then once daily for 16days. Mice were intravaginally inoculated with 5 × 10^4^ *C. albicans* 96,113. Vaginal lavage fluid was collected longitudinally over a period of 18days post-inoculation. Levels of **(A,D)** IL-1β and **(B,E)** S100A8 in vaginal lavage fluid were quantified by ELISA over a period of 18days post-inoculation. **(C,F)** LDH release in vaginal lavage fluid was measured as an indicator of tissue damage. Data represent cumulative results of 2 independent experiments performed with 4–10 animals/group. Data were analyzed by using the unpaired Student’s t test comparing the drug-treated groups with vehicle control group at specific time points. LDH, lactate dehydrogenase.

Given the brief but significant reduction in PMN migration after long-term drug treatment, we assessed the levels of the hallmark inflammatory proteins and LDH in vaginal lavage fluid as additional measures of VVC immunopathology. Results from inoculated mice treated with montelukast or zafirlukast showed similar levels of vaginal IL-1β (Figures 3A,D), S100A8 (Figures 3B,E), or LDH (Figures 3C,F) compared to the vehicle controls. Notably, the inflammatory proteins and LDH levels were not affected by the transient reduction in PMN migration on days 10 (montelukast) and 14 (zafirlukast) post-inoculation (Figure 2B).

### LT Deficiency Does Not Affect *C. albicans* Vaginal Colonization or VVC Immunopathology

**Figure 4:**
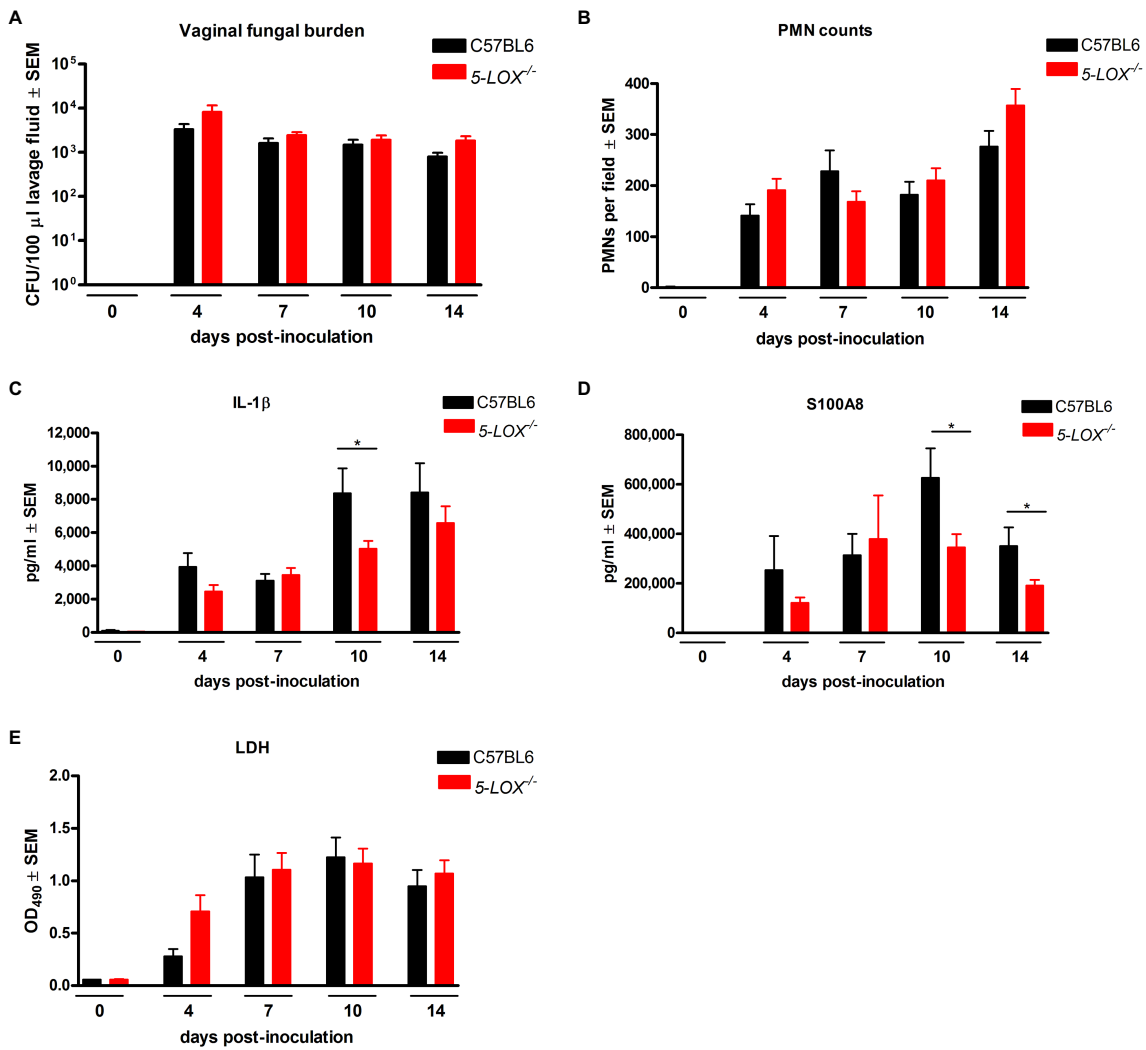
5-lipoxygenase/leukotriene pathway is not required for vaginal immunopathology during VVC. Estrogenized C57BL6 wild-type (C57BL6) or 5-lipoxygenase deficient (*5-LOX^−/−^*) mice were intravaginally inoculated with 5 × 10^6^ *C. albicans* SC5314. Vaginal lavage fluid was collected longitudinally over a period of 14days post-inoculation. **(A)** Vaginal fungal burden was assessed by quantitative plate counts at the indicated time points. **(B)** Vaginal cellular infiltrates were stained by the Pap smear technique and examined by light microscopy at x400 magnification. Number of PMNs were quantified in 5 nonadjacent fields per sampled and averaged. Levels of **(C)** IL-1β and **(D)** S100A8 in vaginal lavage fluid were quantified by ELISA. **(E)** LDH release in vaginal lavage fluid was measured as an indicator of tissue damage. Data represent cumulative results of 2 independent experiments performed with 5–15 animals/group. Data were analyzed by using the unpaired Student’s t test comparing the 5-LOX knockout group with the wild-type control group at specific time points. ^*^p<0.05. CFU, colony-forming unit; PMN, polymorphonuclear leukocyte; LDH, lactate dehydrogenase.

Based on 5-LO as an essential enzyme required for the conversion of arachidonic acid to LTA_4_, the precursor molecule for all effector LTs that retain biological activity in local tissues (Dixon et al., 1990), we employed 5-LO knockout (*5-LOX^−/−^*) mice to determine whether the absence of LT products could modulate the immunopathology. Results showed that *5-LOX^−/−^* mice inoculated with *C. albicans* SC5314 had persistent fungal burden (Figure 4A) and PMN infiltrates (Figure 4B) similar to inoculated wild-type mice. The levels of vaginal IL-1β (Figure 4C) and S100A8 (Figure 4D) in *5-LOX^−/−^* mice were similar as well to wild-type mice with the exception of a moderate, but statistically significant decrease in both mediators at day 10 post-inoculation. There was no difference in LDH release between *5-LOX^−/−^* and wild-type mice throughout the infection period (Figure 4E). Similar results were obtained in *5-LOX^−/−^* vs. wild-type mice inoculated with a vaginal *C. albicans* isolate 96,113 (data not shown). We also confirmed the vaginal presence of LTs in infected wild-type C57BL6 and C3H/HeN mice inoculated with either *C. albicans* strain SC5314 or 96,113; LTB_4_ concentrations were significantly increased in vaginal lavage fluid from inoculated mice compared to negligible levels in uninoculated mice (Supplementary Figures 2A,B). Negligible levels of LTB_4_ were also confirmed in *5-LOX*^*−/*−^ mice (data not shown).

## Discussion

Given the wide use of antifungals that often fail to provide complete clearance and long-term relief for VVC, there remains a need for more effective antifungals or immunotherapies that can alleviate the symptoms of vaginitis. In cases of severe RVVC, constant need for maintenance antifungal therapy leads to negative quality-of-life and significant healthcare burden for affected women. Here, our aim was to determine whether repurposing of two established cys-LT receptor antagonists, montelukast and zafirlukast, as well as a second-generation selective LTB_4_ receptor antagonist, etalocib, could offer a novel immunotherapeutic strategy for long-term symptom relief. Results showed that none of the drugs were able to ameliorate the parameters of VVC immunopathology to any measurable level. Recognizing the dysfunction in PMNs against *Candida* in the vaginal environment (Yano et al., 2017), a reduction in PMNs to any extent by the drugs would not have been expected to modulate vaginal fungal burden, but we did expect a reduction to be reflected in the inflammatory parameters (IL-1β, S100A8, LDH). However, not only were the drugs ineffective, the lack of effects on the immunopathology was further supported by similar immunopathologic parameters in inoculated *5-LOX^−/−^* mice compared to wild-type mice.

Together, these findings were rather surprising given the previous open-label pilot study demonstrating clinical efficacy in maintaining a symptom-free period following a 24-week treatment with zafirlukast and for up to 1year following the final dose (White et al., 2004). In fact, this animal study was borne out of the pilot study so as to inform the design for a larger clinical trial. While recognizing the limited sample size of the pilot study, the discrepancy to the animal study could be the robustness of the mouse model including the requirement for exogenous 17β-estradiol. There is evidence that sex hormones influence 5-LO production and subsequent biosynthesis of LTs, though the effect is predominantly derived from the disparate testosterone levels (Pergola et al., 2008). Importantly, neither 17β-estradiol nor progesterone levels had appreciable effects on LT expression in neutrophils from human blood in response to a variety of stimuli for 5-LO induction (Pergola et al., 2008). Furthermore, a study conducted in mouse zymosan-induced peritonitis revealed similar levels of LTB_4_ and LTC_4_ in peritoneal exudates of females and orchidectomized males compared to sham males that exhibited significantly lower peritoneal LT levels (Rossi et al., 2014). Hence, androgens appear more downregulatory for the 5-LO activity. Taken together, although the animal model is robust, it is unlikely that the estrogen treatment adversely affected the action of the LTRA drugs or LT biosynthesis.

Taking into account a strong PMN response as the chief mediator of VVC immunopathology, etalocib presented a strong potential owing to its selective action on LTB_4_-mediated neutrophil responses (Ford-Hutchinson et al., 1980; Jackson et al., 1999; Silbaugh et al., 2000; Chen et al., 2006). To our disappointment, etalocib failed to dampen the PMN response during infection. Due to its poor solubility, however, our experimental concentrations were limited to a fairly low range (≤1mg/kg). It is possible that etalocib could exert an anti-inflammatory effect on the vaginal mucosa at higher concentrations, possibly by use of a salt formulation with increased solubility (not commercially available; Kuwabara et al., 2000; Silbaugh et al., 2000). Interestingly, although a study conducted in patients with psoriasis indicated that etalocib was safe and well-tolerated (Pelt et al., 1998), the development of the drug for treatment of inflammatory indications was discontinued. A more recent study concluded that etalocib was not effective in preventing recurrent psoriasis (Mommers et al., 2000).

Montelukast and zafirlukast are currently approved medications indicated for the prevention and treatment of chronic bronchial asthma (Choi and Azmat, 2021) and have been investigated for other inflammatory conditions such as allergic rhinitis, inflammatory bowel disease, chronic obstructive pulmonary disease (COPD), and urticaria (hives; Mahgoub et al., 2003; Riccioni et al., 2007; Khan and Lynch, 2012). Cys-LT-mediated pathology is commonly manifested as increased vascular permeability and mucus production, mainly by activation of mast cells, eosinophils, and basophils that are not part of VVC immunopathology (Peters-Golden and Henderson, 2007; Theron et al., 2014). However, the transient reduction in PMN migration was encouraging and prompted us to further verify the effect of the drugs in vaginal inflammatory markers (IL-1β, S100A8) and tissue damage (*via* LDH). Unfortunately, these other parameters could not support any measurable reduction in PMNs indicative of any efficacy. Given no firm evidence of montelukast and zafirlukast efficacy *in vivo* despite employing optimal or the highest concentrations possible, together with the possibility of not achieving an effective dose of etalocib, results from the drug studies remained somewhat insufficient to reach an absolute conclusion on potential for clinical use. Since 5-LO is essential for the biosynthesis of all LT derivatives, *5-LOX^−/−^* mice are resistant to LT-associated inflammatory conditions, such as PMA-induced skin edema and immune complex peritonitis (i.v. ovalbumin followed by i.p. anti-ovalbumin IgG), in which *5-LOX^−/−^* mice exhibited significantly reduced PMN infiltrates compared to wild-type mice (Chen et al., 1994). Hence, the *5-LOX^−/−^* mice represented a strong parallel design to aid the conclusions of the drug studies. Results revealed that *5-LOX^−/−^* and wild-type mice exhibited similar outcomes in all immunopathologic parameters of vaginitis. In addition, similar results were obtained regardless of the *C. albicans* strains (a laboratory strain vs. a vaginal isolate) at varying inocula, confirming a lack of variables potentially introduced by fungal factors. Moreover, the *5-LOX^−/−^* mice in the C57BL/6 background had a normal PMN response comparable to our standard design using C3H mice. Thus, we showed similar results of LT deficiency/antagonism that were reproducible in two different strains of mice with distinct MHC haplotypes. While this indeed represents strong evidence overall, the literature suggests regulation of pro-inflammatory cytokines by 5-LO in response to pathogens, as well as interaction of S100A8/A9 with arachidonic acid and its metabolites. A transient reduction seen in the levels of IL-1β and S100A8 could be due to deletion of *5-LOX* that may have had an indirect impact on protein expression or secretion. Support for this includes (i) reduced systemic cytokine production including IL-1β in *5-LOX^−/−^* mice in a cecal ligation and puncture (CLP)-induced peritonitis model (Monteiro et al., 2014), (ii) S100A8/A9 sequestration by arachidonic acid that possibly becomes accumulated due to a lack of active 5-LO (Kannan, 2003), or (iii) downregulation of S100A8 expression in *5-LOX^−/−^* mice in the absence of LTA_4_ which was shown to scavenge S100A8/A9 proteins for protection from hydrolytic degradation (Rector and Murphy, 2009). While these alternative explanations are plausible in our model, all data realistically point to LTs being dispensable to VVC immunopathogenesis. Thus, agents that target LTs are not likely viable options as therapeutics to reduce symptomatic VVC.

Despite the contradictory results between the mouse model and open-label pilot study (White et al., 2004), there still may be some motivation for a phase III clinical trial with zafirlukast in cases of VVC or RVVC. Since vaginal PMN infiltrates were not evaluated in the pilot study, as in most clinical settings, the magnitude of vaginitis symptoms can become highly subjective or variable between women. Hence, a trial that includes multi-parameter examination of VVC/RVVC symptomatology *via* vaginal PMN infiltrates and inflammatory mediators could serve as the most reliable indicator of the immunopathology and confirmatory correlates to general signs and symptoms. Additionally, although we feel that RVVC is an exacerbated immunopathogenesis under similar mechanisms to VVC, results from our model that only evaluated acute VVC should not be presumed to apply to RVVC without further experimentation.

In summary, we have demonstrated that pharmacological inhibition or genetic deletion of LT production had no influence on the outcomes of fungal loads and all inflammatory parameters of vaginitis in the mouse model. These data therefore suggest that LTs are dispensable to VVC immunopathology. Based on the detectable and increased presence of vaginal LTB_4_ during acute VVC shown here, as well as during experimental vaginal *Trichomonas vaginalis* infection (Eida et al., 2015), it will be interesting to determine the mechanisms of LT production and receptor expression, both locally and systemically, during vaginal infection. Unfortunately, the assessment of LTs (locally or systemically) in the presence of the drugs is not informative since the drugs act as receptor antagonists and not synthesis inhibitors. Irrespective though it will be interesting to identify what pathways are involved in PMN migration under targeted antagonism/inhibition. This together with current strategies to overcome/rescue the PMN dysfunction (neutrophil anergy) in order to achieve adequate vaginal antifungal activity will be critical to reducing the immunopathology and provide relief to the large number of women affected by VVC/RVVC.

## Data Availability Statement

The raw data supporting the conclusions of this article will be made available by the authors, without undue reservation.

## Ethics Statement

The animal study was reviewed and approved by Institutional Animal Care and Use Committee (IACUC) of the LSU Health-New Orleans.

## Author Contributions

PF conceived and designed the experiments. JY performed the experiments. FW provided mouse breeders. JY and PF analyzed the data and wrote the manuscript. DW, AS, and FW edited the manuscript. All authors contributed to the article and approved the submitted version.

## Funding

This work was supported by the LSUHSC Foundation. The Foundation had no role in study design, data collection, and interpretation or the decision to submit the work for publication.

## Conflict of Interest

The authors declare that the research was conducted in the absence of any commercial or financial relationships that could be construed as a potential conflict of interest.

## Publisher’s Note

All claims expressed in this article are solely those of the authors and do not necessarily represent those of their affiliated organizations, or those of the publisher, the editors and the reviewers. Any product that may be evaluated in this article, or claim that may be made by its manufacturer, is not guaranteed or endorsed by the publisher.

## Supplementary Material

Supplementary Figure S1 Schematics of the 5-lipoxigenase/leukotriene pathway and pharmacologic receptor antagonists. Enzymatic metabolism of arachidonic acid induced by inflammatory stimuli results in production of a variety of eicosanoids. Metabolism via the LO pathway by the 5-LO enzyme leads to the synthesis of LTs. Receptor binding of LTs on target cell-surface receptors activates a wide range of proinflammatory reactions, which can be pharmacologically blocked by cysteinyl LT (LTC_4_, LTD_4_, and LTE_4_) receptor antagonists, Zafirlukast and Montelukast, or LTB_4_ receptor antagonist, Etalocib.

Supplementary Figure S2 LTB_4_ is secreted into the vaginal lumen during *C. albicans* vaginal infection. Estrogenized (A) C57BL/6 and (B) C3H/HeN mice were intravaginally inoculated with 5 x 10^6^ *C. albicans* SC5314 (A) or 5 x 10^4^ *C. albicans* 96,113. Vaginal lavage fluid was collected longitudinally over a period of 14 days post-inoculation. Levels of LTB_4_ in vaginal lavage fluid were quantified by ELISA. Data included measurements from 4 animals/group and were analyzed by using the unpaired Student’s t test comparing the pre- vs. post-inoculation samples at specific time points. LTB_4_, leukotriene B_4_. **p < 0.01; ***p < 0.001.
